# ConGen—A Simulator-Agnostic Visual Language for Definition and Generation of Connectivity in Large and Multiscale Neural Networks

**DOI:** 10.3389/fninf.2021.766697

**Published:** 2022-01-07

**Authors:** Patrick Herbers, Iago Calvo, Sandra Diaz-Pier, Oscar D. Robles, Susana Mata, Pablo Toharia, Luis Pastor, Alexander Peyser, Abigail Morrison, Wouter Klijn

**Affiliations:** ^1^Simulation and Data Lab Neuroscience, Jülich Supercomputing Centre, Institute for Advanced Simulation, JARA, Forschungszentrum Jülich GmbH, Jülich, Germany; ^2^Department of Computer Science and Computer Architecture, Lenguajes y Sistemas Informáticos y Estadística e Investigación Operativa, Rey Juan Carlos University, Madrid, Spain; ^3^Center for Computational Simulation, Universidad Politécnica de Madrid, Madrid, Spain; ^4^DATSI, ETSIINF, Universidad Politécnica de Madrid, Madrid, Spain; ^5^Institute of Neuroscience and Medicine and Institute for Advanced Simulation and JARA BRAIN Institute I, Jülich Research Centre, Jülich, Germany; ^6^Computer Science 3 - Software Engineering, RWTH Aachen University, Aachen, Germany

**Keywords:** multiscale simulation, large scale simulation, visual language, neural networks, connectivity generation, connectome

## Abstract

An open challenge on the road to unraveling the brain's multilevel organization is establishing techniques to research connectivity and dynamics at different scales in time and space, as well as the links between them. This work focuses on the design of a framework that facilitates the generation of multiscale connectivity in large neural networks using a symbolic visual language capable of representing the model at different structural levels—ConGen. This symbolic language allows researchers to create and visually analyze the generated networks independently of the simulator to be used, since the visual model is translated into a simulator-independent language. The simplicity of the front end visual representation, together with the simulator independence provided by the back end translation, combine into a framework to enhance collaboration among scientists with expertise at different scales of abstraction and from different fields. On the basis of two use cases, we introduce the features and possibilities of our proposed visual language and associated workflow. We demonstrate that ConGen enables the creation, editing, and visualization of multiscale biological neural networks and provides a whole workflow to produce simulation scripts from the visual representation of the model.

## 1. Introduction

The brain has a multilevel organization, with anatomical and dynamic features spanning orders of magnitudes. Understanding the nature of its components and how they are connected with each other is critical to unraveling this complexity (Evanko and Pastrana, 2013; Morgan and Lichtman, 2013; Peyser et al., 2019), for both healthy and diseased brains (e.g., Chen et al., 2021). Indeed, connectivity is an essential aspect defining the functionality at all organizational scales of the brain (Sporns et al., 2005).

The 21st century has seen multiple interdisciplinary research initiatives initiated to address this important topic (Collins and Prabhakar, 2013; Markram et al., 2015); however, despite advances and efforts toward standardization in this field (Gadde et al., 2012; Gorgolewski et al., 2016), there is no consistent way to represent, visualize, explore, and generate connectivity for simulation or analysis across different scales. Consequently, developing the tools to support investigations of multiscale functional organization remains an open challenge.

A central method in such investigations is numerical simulation of the brain. Existing simulation engines capture the brain behavior at different levels of detail: detailed multi-compartment simulations (e.g., Arbor—Akar et al., 2019; Abi Akar et al., 2021, Neuron—Carnevale and Hines, 2006), point neuron simulations (e.g., NEST—Jordan et al., 2019, Brian—Stimberg et al., 2019) or whole brain level simulations (e.g., The Virtual Brain—TVB Sanz Leon et al., 2013). By exploiting high performance computing we are now able to simulate large scale networks as well as those which represent the brain at different scales simultaneously. However, the absence of methods to create, and explore complex connectivity in these types of networks limits investigations in the relationships between connectivity and function.

Creating a framework that enables simulation of large and multiscale models of heterogeneous neuron populations is only possible by making use of well-defined interfaces, either new or existing. These interfaces must allow weak coupling between software systems and the necessary front ends to interact with them, but at the same time be able to leverage the native functions of the simulation engines and their inherent scaling capabilities. The development of tools and standards which take advantage of these interfaces will additionally enable the comparison of performance and function metrics between different simulation engines. This will allow the formulation of more robust scientific conclusions and move the field of computational neuroscience forward. The implementation of easy to use and flexible tools for benchmarking different simulation tools remains a critical and still unfulfilled requirement by the neuroscience community.

This work focuses on the design and implementation of ConGen, a framework that facilitates the generation of connectivity in large neural networks using a new symbolic visual language capable of representing the model at different structural levels. This symbolic language is specifically designed to provide researchers with a tool to represent and explore the connectivity in models of multiscale and large scale networks. ConGen provides an agnostic way to represent models and later instantiate them with specific simulation frameworks. The connectomes represented in the visual language can be exported to the standardized network representation format NeuroML (Gleeson et al., 2010). These descriptions can be used directly by simulation engines which support the format. ConGen adds functionality with a back end, also interfaced with NeuroML.

The ConGen back end enables interfacing with efficient connection generation approaches (e.g., Djurfeldt et al., 2014) and allows users to launch simulations using the simulation engines' native scaling capabilities. For convenience, the ConGen back end encapsulates a set of basic templates allowing users to generate the connectivity using the standard description. The ConGen back end also includes a number of thin simulator-specific interfaces, enabling basic launching, i.e., using default model parameters, on the target simulator. Users can edit and extend the thin simulator-specific scripts in the ConGen back end to define their own simulation and model parameters. Currently the ConGen back end supports execution of the NEST and TVB simulators, as well as the generation of EBRAINS co-simulation model scripts ready for execution by external tooling. However, due to its modular design it is possible to easily extend the back end to support other simulators.

With this work we provide a bridge between a simple, yet expressive, visual language (see section 2.1.1) embedded into a simulator-agnostic graphical interface, simulation frameworks, and high performance computing infrastructure. By providing a language to describe connectivity in a simulator-agnostic way, ConGen also represents a new platform to assist benchmarking using a generic description of networks based on model description standards. As such, ConGen helps to address the unfulfilled requirement for an easy to use and flexible tool for benchmarking.

This paper is structured as follows: first, we present the state of the art in connectivity visualization and representation techniques, as well as standards for network description. Then, we describe the front end which implements the symbolic visual language: ConGen. Afterwards, we discuss the implementation of the back end which takes the standard output representation from the visual language and translates it into a model instance in one or multiple simulators. In the results section we show two use cases for this framework. The first refers to the well-known model of the cortical mircrocircuit by Potjans and Diesmann (2014) and the second is a multiscale model which combines a simulation in TVB and NEST. Finally, we discuss the use cases and the current limitations of the framework, provide some conclusions, and point toward future directions.

### 1.1. State of the Art

#### 1.1.1. Visual Representations of Connectivity

Connectivity matrices have long been used as a way to represent connections in the brain. For example, the work of Rubinov and Sporns (2010) presents a Matlab Toolbox intended to generate connectomes at the scale of brain regions using this type of representation. Here, binary entries in the matrix indicate the presence or absence of connections; real-valued entries can be used to represent magnitudes regarding correlational or causal interactions. Although the authors state that the neuroimaging methods available for them were unable to directly detect anatomical or causal directionality, the matrices produced using the Toolbox can incorporate this information if it is available. Mijalkov et al. (2017) created another Matlab Toolbox that allows the user to create visualizations mainly based in connectivity matrices derived from different neuroimaging modalities with the aim to study large scale brain connectivity applying techniques from graph analysis theory.

An alternative representation of connectivity is given by a Connectivity Pattern Table (CPT), a 2D schematic and compact representation intended to shown the spatial structure of connections as well as their strength, proposed by Nordlie and Plesser (2010). The main features of CPTs are a clutter-free presentation of connectivity, the ability to represent connectivity at several levels of aggregation and a high information contents regarding the spatial structure of connectivity.

Other approaches have focused on morphologically detailed connectivity. For example, NeuroLines (Al-Awami et al., 2014) is a multiscale abstract visualization technique for the analysis of neurites and their connections. Here, each neurite is represented as a tree structure based on 3D data of their morphology. Once a synapse is selected, all other synapses linked to the same neurites are visually highlighted for contextual information. In related work, Böttger et al. (2014) developed an edge bundling method which depicts clear and high-resolution pictures of functional brain connectivity data across functional networks in the 3D brain space.

Additional tools address MEG/EEG data import and pre-processing. NeuroPycon (Meunier et al., 2020) is a Python toolbox for the visualization of connectivity analysis in MEG sensors. The visualization is built from the sensor-level connectivity matrix obtained from the computation of the coherence among MEG sensors in alpha band. The colors of the connectivity edges indicate the strength of the connection, and the node size and color represent the number of connections per node. Similarly, Espinoza-Valdez et al. (2021) presented a 3D visualizer of the brain connectivity for EEG data. The selection of electrodes is performed in a dynamic way; graph theory is then applied to characterize brain connectivity in 3D images.

Finally, Fujiwara et al. (2017) introduced a visual analytics system to enable neuroscientists to compare networks. The system provides visual tools for comparison at both individual and population levels. The main visualization techniques they use are based on representations of connectivity and node-linkage matrices (both 2D and 3D).

#### 1.1.2. Abstract Representations of Connectivity

Unfortunately, often the descriptions of network model connectivity do not adhere to any standards (Nordlie et al., 2009). Model definitions rely on a combination of complex text descriptions, pieces of pseudo-code or simulator-specific code, tables, and connectivity patterns without formal definitions. Consequently, ambiguities in the model description make it difficult to independently reproduce the network, or port it from one simulation environment to another (Pauli et al., 2018).

To provide a formal standard that can be used to convey the connectivity of a model, not only in written text and formulas, but also among neuronal simulators, Djurfeldt (2012) developed the Connection Set Algebra (CSA): a mathematical representation of connections between populations of neurons based on set algebra. With this abstract formalism, a connectivity pattern can be defined independently of the implementation by the various simulators. This independence is an important aspect of the modular nature of CSA, allowing it, in principle, to be used in combination with any simulator. The connection with the simulators is formalized in the Connection Generation Interface (CGI; Djurfeldt et al., 2014). The CGI allows the simulator to query connections from the linked connection generator. Both the simulator and the connection generator need to implement the interface.

#### 1.1.3. Standardized Network Model Descriptions

A number of domain languages exist to describe networks at different scales, notably PyNN (Davison et al., 2009), NeuroML (Gleeson et al., 2010), and NineML (Raikov et al., 2011). PyNN is a Python based simulator-independent language. It supports modeling at multiple levels of abstraction. The instruction set of each simulator and PyNN code can be mixed, so models described in PyNN can still access features specific to individual simulation engines. Importantly for our work, PyNN implements the CGI, which allows connection generation using the CSA. During the development of ConGen, PyNN did not support NeuroML, but a NeuroML file export for networks generated with PyNN has since been added. While PyNN enables easier interfacing with various simulators, it has been designed primarily as a scripting tool. No visual tools are available for PyNN, instead network creation follows procedural instructions.

NeuroML is a simulator-independent XML-based formalism that is supported by a variety of neuroscience tools and supports a more biophysically detailed level of modeling than PyNN. The standard consists of three levels, which are built hierarchically and provide a standard for describing morphologically detailed neurons, spiking neurons and populations of neurons. The most recent version of NeuroML (NeuroML2) combined with LEMS (Cannon et al., 2014) has been developed in order to be able to represent both network structure and model dynamics in a standardized and domain specific fashion.

Finally, NineML is an XML-based modeling language similar to NeuroML formalized in an XML Schema Definition (Raikov et al., 2011). Its primary focus is on definitions on the network level, such as populations and connections; as a consequence of this focus it lacks many of the detailed elements present in NeuroML, e.g., biological cell structures of neurons and synapses.

The computational neuroscience community needs to further use and define standards in order to promote reproducibility and robustness of results. With this in mind, efforts like Open Source Brain (Gleeson et al., 2019) try to integrate graphic user interfaces, model description languages and simulation engines into a cohesive effort to simulate the brain.

#### 1.1.4. Simulation Engines

Simulators are an important tool in computational neuroscience. Simulation engines enable the creation and simulation of models at different scales. They typically provide a language, usually a scripting language, for the user to access the simulator's functions.

Some common spiking neuron simulation tools are NEURON (Carnevale and Hines, 2006), NEST (Jordan et al., 2019), and BRIAN (Stimberg et al., 2019). For a detailed comparison on these and other simulators see Tikidji-Hamburyan et al. (2017). Of the three simulators, NEURON is the one with the longest history and largest user community. Arbor (Akar et al., 2019) is a new simulation framework, developed in the context of the HBP, at the morphologically detailed scale and is designed to take full advantage of new computing architectures and reach high scalability. While NEURON and Arbor are used for detailed cell models, NEST is used to simulate primarily point neurons, and multi compartment neurons with up to three compartments. NEST is optimized for simulations of large scale networks—including up to hundreds of millions of neurons and their synapses—on high performance computers while still having great performance on smaller devices. BRIAN supports simulations of both detailed and large scale networks with a focus of separating model definition and simulator implementation details. For ease of use all these simulators have implemented Python interfaces, or can be controlled using a simulator specific language [e.g., PyNEST (Eppler et al., 2009) for NEST].

Simulations of the whole brain are also possible at a coarse resolution. For example, The Virtual Brain is a simulation framework which allows the representation of the brain using neural mass models and simulate them to generate synthetic Electroencephalography (EEG), Magnetoencephalography (MEG), or Blood Oxygen Level Dependent (BOLD) signals. Another emerging simulator at the whole brain scale level is neurolib (Cakan et al., 2021). Similar to TVB, neurolib provides the end user with a variety of neural mass models, the ability to create networks based on empirical connectivity data and generate simulated signals which can be optimized using parameter fitting methods against empirical data.

## 2. Methods

This section describes the two components of the proposed framework: the description of the multiscale connectivity using a symbolic visual language, and the translation of the generated models into simulator-specific instructions.

The ConGen front end provides end users with a standardized description of their models in NeuroML. This description can be directly used by simulation engines which support this standard. For convenience, the ConGen back end encapsulates a set of basic functionality allowing users to read the NeuroML file and generate the connectivity. The ConGen back end includes simulator specific thin interfaces, allowing for basic launching on the target simulator. To increase the compatibility of ConGen to multiple simulators, we have extended the NeuroML scheme used to define the models. Illustrating the flexibility of the toolset, one of our use-case expands on the standard NeuroML interface with new functionality. We show that new simulators and functionality can be added by adapting the ConGen back end. Users can also directly interact with the output of the ConGen front end in NeuroML format to create more complex simulations with detailed model specifications which go beyond basic configuration and connectivity definition e.g., cell model specific parameters, pre and post- processing of input and output data, etc.

### 2.1. Visual Front End for Connectivity Generation

ConGen facilitates the creation, editing and visualization of multiscale neural networks. Connections can be created and visualized at the desired level of abstraction, and mechanisms for the propagation and aggregation of connectivity along the hierarchy are provided. This approach allows the researcher to generate large scale scenarios capturing global behavior and local details at the same time.

The ConGen front end has been integrated into Neuroscheme (Pastor et al., 2015), a visual framework to guide exploration and knowledge extraction from complex neural scenes. Neuroscheme allows the creation of domains that define the set of elements that conform a neuronal scene. For example, Neuroscheme includes the cortex domain, which provides elements corresponding to the organizational levels of column, minicolumn and neuronal cell, as well as defining the properties associated with each element. ConGen has been conceived and developed as a new domain within Neuroscheme, defining a new set of abstract neural elements (i.e., not corresponding to specific brain areas) and connections between them in order to represent models of large scale and multiscale neural networks.

**Figure 1:**
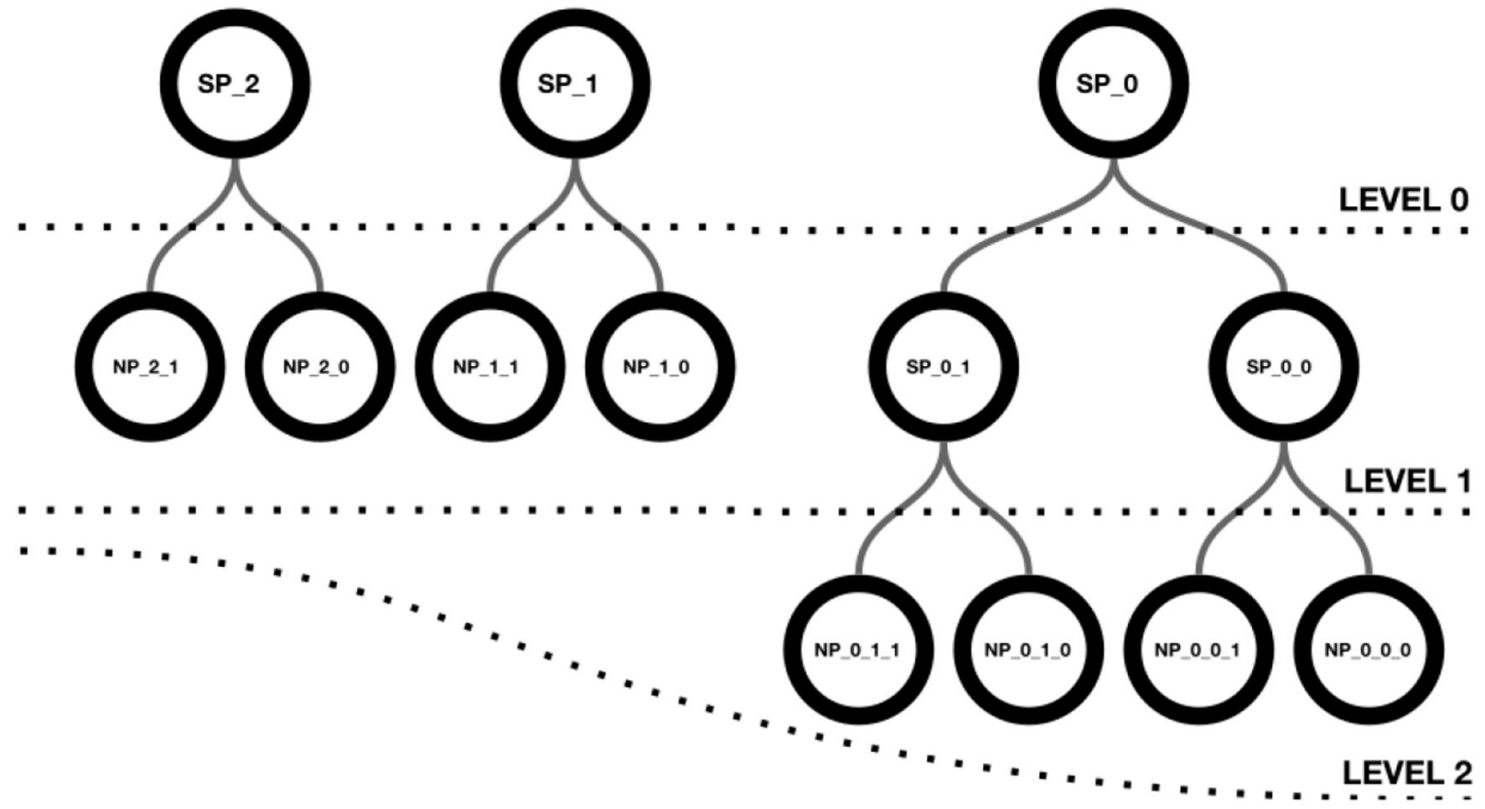
Hierarchical structure of a scene. Level 0 shows three superpopulations that group either populations or descendant superpopulations, as shown in level 1. Superpopulations SP_0_0 and SP_0_1 contain two neuron populations each, as depicted in level 2.

Neuroscheme offers an environment with multiple views where different representations of the data can be visualized in a coordinated manner. In this way, abstract views can be combined with accurate representations of cellular anatomy. The iconic view of a circuit provides a global, simplified view with summarized or aggregated information, while the realistic view provides all details of the neuronal anatomy and spatial distribution. ConGen has been designed to act as a front end for interactive visual definition of neural connectivity, thereby facilitating the creation and manipulation of neural circuit models. Following a top-down approach, ConGen enables the creation of a hierarchy of super-populations and populations and the specification of their connections by establishing the necessary connectivity parameters. Populations constitute the leaves of the hierarchy and grouping them together gives rise to a superpopulation. In turn, superpopulations can be grouped iteratively, also giving rise to hierarchical superpopulations. Figure 1 shows a hierarchy of superpopulations and populations. Our approach to interaction and visual representation emphasizes simplicity, depicting views using easy symbolic representations. The models so created can be exported using an extended version of NeuroML for further simulator-specific translation. The following subsection details the operations supported by ConGen.

#### 2.1.1. Creation and Parameterization of a Hierarchical Network Structure

**Figure 2:**
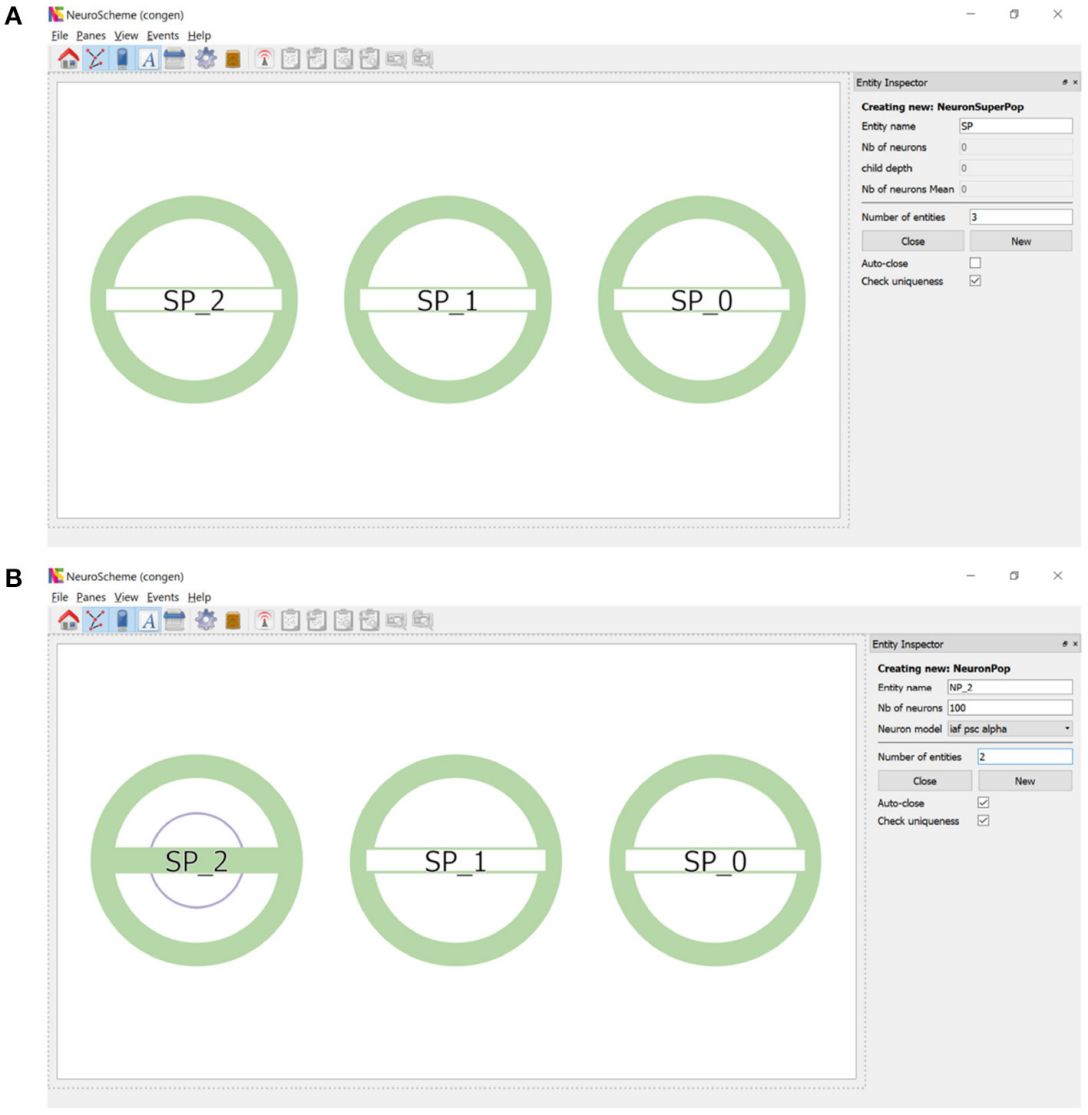
Creation of super-populations and populations. **(A)** The panel on the right sets the name root of the super-populations (SP in this example) and the number of entities (three in this example) to be created. **(B)** Right clicking on SP_2 allows the creation of descendant neuron populations. The panel on the right sets the name root of the populations (NP_2 in this example) and the number of entities (two in this example) to be created.

ConGen supports the creation of a neural scene and its connectivity by providing an interface that visually displays the created entities and relationships. Each entity will be represented by a circular shape. Entities of the same type will share the same color (superpopulations, populations, inputs and outputs). The number of inner circles will represent the number of descending levels of a superpopulation and the filling of the horizontal bar will be proportional to the number of neurons in each grouping. By simply right-clicking with the mouse, a context menu appears allowing entities to be created and hierarchically structured. To create one or more super-populations, the user simply sets the number of entities to be created, their name and the other configurable parameters. Figure 2A shows the super-population creation panel as well as the visual representation of the three super-populations created. Figure 2B illustrates the creation of two populations of neurons within the super-population SP_2. Note that the visual representation of the super-population SP_2, compared to its appearance in Figure 2A, now reflects the existence of a hierarchical descendant level (presence of an inner circle) and the number of neurons in the descendant populations (green filling of the horizontal bar).

**Figure 3:**
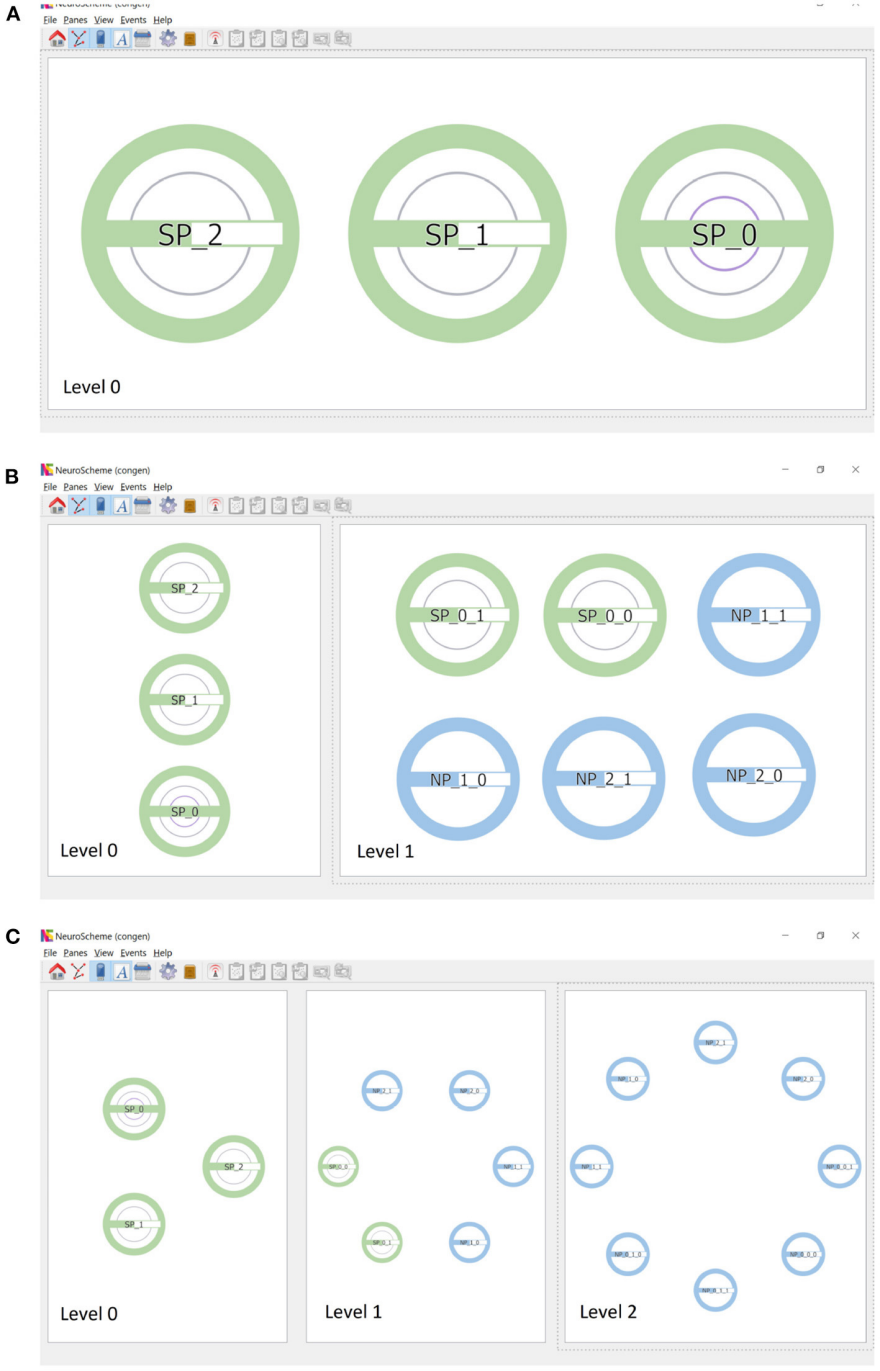
Creation of scenes in ConGen. **(A)** A scene where SP_0 has two hierarchical descendant levels (indicated by the two inner rings) while SP_1 and SP_2 have one hierarchical descendant level each. The green filling of the horizontal bars indicates that SP_1 and SP_2 have the same number of neurons while SP_0 has twice the amount. **(B)** Super-populations can be expanded to visualize the next hierarchical level. Left panel: the three super-populations in a collapsed view. Right panel: The three super-populations have been expanded to show their direct children. **(C)** The panels show the three hierarchical levels of the scene simultaneously. Left: Neuron super-populations at the highest level of abstraction. Middle: The hierarchical entities tree displayed at depth level 2. Right: All entities have been drilled down to show the lowest level of abstraction. Icons have been arranged in a circular layout for convenience for connectivity creation.

Continuing the procedure outlined above, the hierarchy initially shown in Figure 1 can be easily created. Figure 3A shows this scene depicted at level 0, composed of three super-populations (SP_0, SP_1 and SP_2). SP_0 in turn contains two child super-populations (SP_0_0 and SP_0_1); each of them, as well as SP_1 and SP_2, containing two populations of neurons. The super-populations can be expanded to show their children, either in the same panel or in a different panel; Figure 3B shows the result of expanding all super-populations in a different panel, thus allowing the scene to be visualized at two levels of abstraction simultaneously (level 0 on the left, and level 1 on the right). Similarly, the SP_0_0 and SP_0_1 super-populations can be expanded into a further panel, depicting the scene at the lowest level of abstraction in the right panel of Figure 3C.

**Figure 4:**
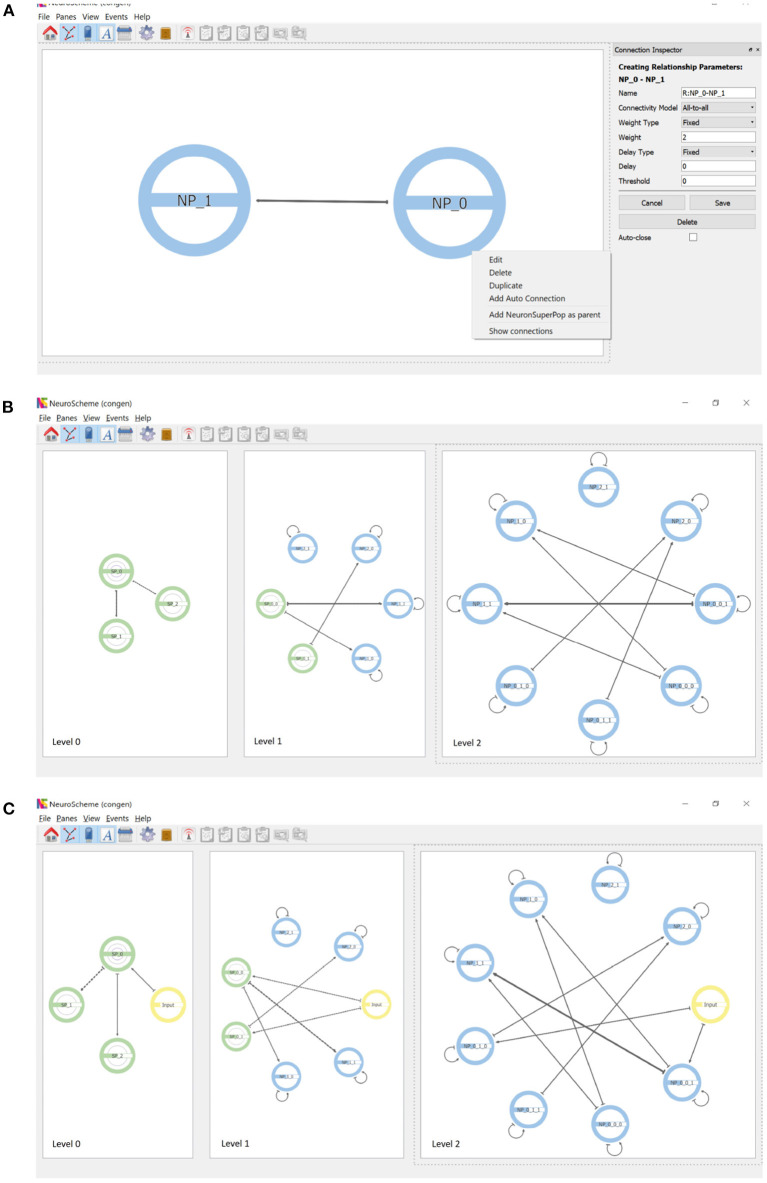
Connections and inputs. **(A)** Connections are created by dragging with the mouse from the source to the target population. The panel on the right shows the parameterizable features. Auto-connections can be created through the context menu that appears when right-clicking on a population. **(B)** Connectivity simultaneously displayed at three levels of abstraction. Note the connections of superpopulations represent the aggregation of the connections of their descendant populations. **(C)** Inputs can be created as entities that are external to the hierarchy. Note that inputs appear at every level of abstraction.

Connections are created by dragging with the mouse from the source population to the target population. Figure 4A shows the parameterization options of the connections as well as the context menu that allows auto-connections (i.e., connections of a population to itself) to be added. Each connection is represented by an arrow whose thickness is proportional to the strength of the connection. Since the views shown in the different panels are coordinated, the connections created at the lowest level of abstraction are reflected in an aggregated way in the panels showing the scene at higher levels of abstraction, as shown in Figure 4B.

In addition to neuron populations, input and output entities can be created. These entities are external to the hierarchy. Input entities stimulate one or more populations of neurons. An input entity is connected to its target population analogously to the connections between populations. Figure 4C shows the result of including an input connected to example populations NP_0_0_1 and NP_0_1_0; note that input entities appear at all levels of abstraction. Output entities, such a measurement devices, can receive a connection from one or more populations of neurons.

### 2.2. From Visual Representation to Simulation

**Figure 5:**
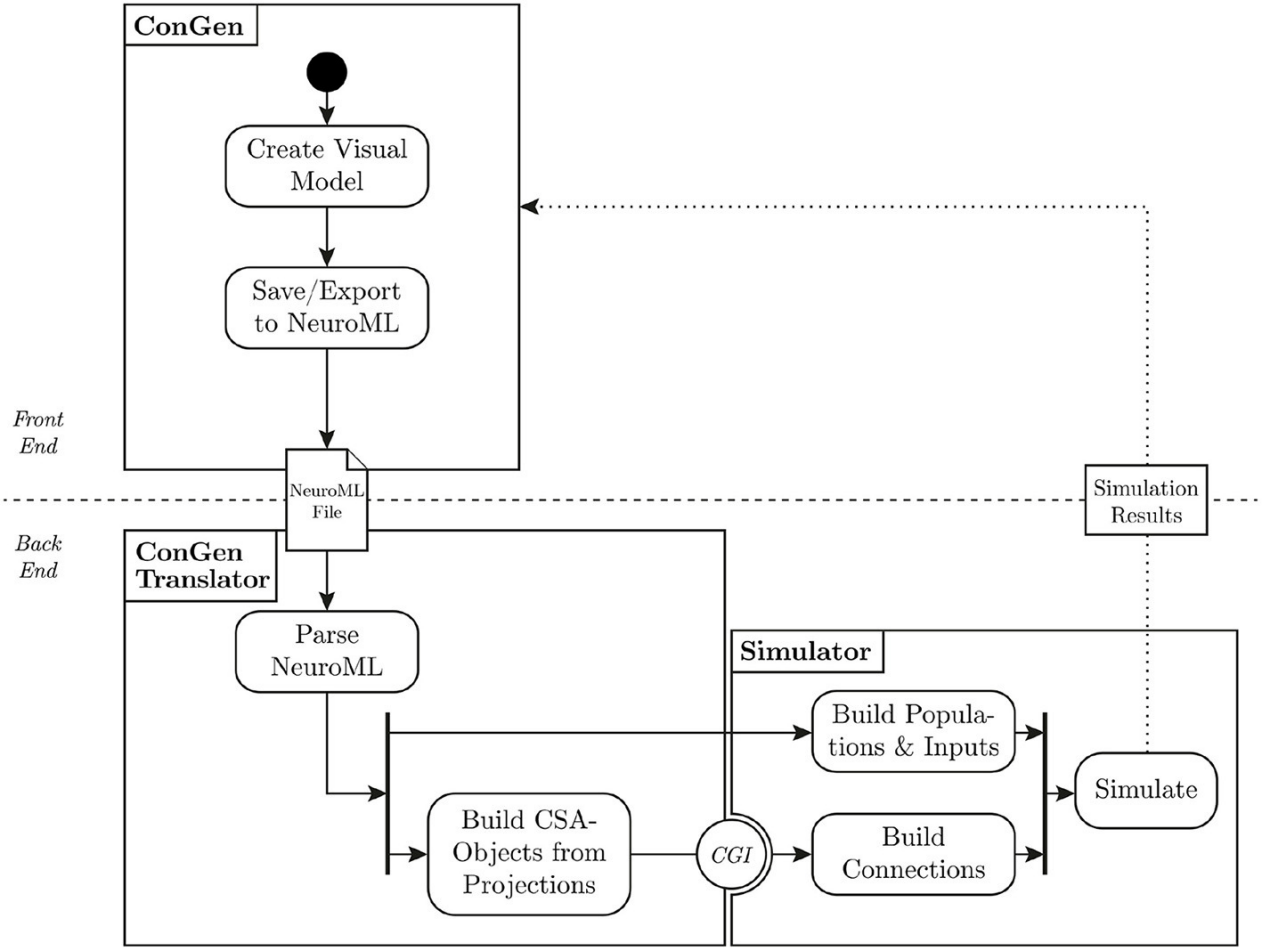
Flow of data from visual representation to simulation. The user creates a model in ConGen and exports it as a NeuroML file. The translator parses the NeuroML file and converts the connectivity into the Connection Set Algebra. The populations and inputs are built directly in the simulator. Using the Connection Generation Interface (CGI), connections are generated from the connection generation library and passed to the simulator, which can then start the simulation. A future extension of this workflow will allow simulation results to be processed and passed back to ConGen (dotted line).

In this section, we introduce the back end of ConGen, which is used to generate the hierarchical neural network models and interact with the simulation engines (see division of front end/back end in Figure 5). The ConGen front end has to serialize the model expressed in ConGen's graphical language by some means. Here, we make use the pre-existing NeuroML standard rather than developing a new declarative language to achieve this goal. Any simulator that supports NeuroML can be considered a potential execution target.

The back end of ConGen consists of a modular translator system. Its purpose is to translate the NeuroML descriptions of the models created in the GUI described in the previous section into simulator-specific code. It is important to highlight that the ConGen back end is not a simulation engine but is able to call functions from different target simulators using the simulator interface. The translation system was designed with the following technical requirements in mind:

1. The translation system should enable the simulation of a model defined in the ConGen GUI by a supported simulator
2. The simulation should be able to be performed in different simulators
3. Adding support for a new simulator should require a low development cost
4. The translation system should be functionally separate from the ConGen GUI
5. The overhead of the system should be low: performance should be close to that of using the simulator directly.

The back end of ConGen is designed to be separate from the GUI (technical requirement 4). This separation allows the front end and back end to be used as independent modules. For example, the front end can run on a desktop computer, while the back end runs and then executes the simulations on a high performance computing system. This modular design also allows individual components to be easily maintained or replaced (technical requirement 3). A modular design requires careful construction of interfaces and data exchange; these are illustrated in Figure 5, which shows the overall flow of data from the visual tool to the simulator.

#### 2.2.1. Using NeuroML

The main data exchange between the front end and the back end is via NeuroML: ConGen serializes its visual models to NeuroML files for storage or data exchange. This separation through a common data standard allows the ConGen translator to be entirely independent of the GUI. ConGen uses NeuroML version 1.8.2, which allows networks, layers, and connections to be represented by XML files. At the time that the back end was developed, NeuroML version 2 was still in development. For this reason, the work presented here is based on version 1.8.2, but will be ported to version 2 in the future.

The structure and validity of files is defined by XML schemas, which allows extensions of the described file format. To increase the compatibility of ConGen to multiple simulators, we have extended the NeuroML scheme used for translation. These additions include spatial connectivity and synapse parameter distributions. All changes are listed in the Supplementary Material (see section Changes to NeuroML).

#### 2.2.2. The ConGen Back End

After a visual model has been saved as a NeuroML file, the file can be used as an input to the ConGen translator. The translator parses the defined network structure, translates the layer and connectivity information, and generates simulator-specific instructions. The translator and the subsequent simulation can be called either independently or invoked directly by ConGen. In the following, we describe the workflow resulting in a set of simulator specific commands which enable the model simulation using specific simulation engines, and how ConGen can be extended to support new simulators.

First, the ConGen back end parses the NeuroML file for translation. XML tags correspond to a Python class, as shown for the example of connectivity patterns in Table 1. The parser first reads the populations, then the projections, and lastly the inputs, outputs, and translators. If the model is to be simulated by different simulators at different scales, the back end splits the model into scale-specific sub-models. After the model has been parsed successfully, all string references between objects are replaced with object references. Any errors present in the file (schema mismatch, undefined references) are raised as an exception.

**Table 1:** XML connectivity pattern tags and their corresponding Python classes and CSA structures.

**NetworkML tag**	**Python class**	**CSA structure**
< all_to_all/>	AllToAll	csa.full
< one_to_one/>	OneToOne	csa.oneToOne
< fixed_probability/>	FixedProbability	csa.random(p)
< per_cell_connection/>	PerCell	csa.random(fanIn=n)
< gaussian_connectivity_2d/>	GaussianSpatialConnectivity	gaussian(sigma)

*The Gaussian spatial connectivity has to be combined with CSA's random operator, which samples from the distribution*.

Connectivity patterns are represented by the ConnectivityPattern class, which may be subclassed when adding new types of connectivity patterns. When an object of this class is created, the connectivity patterns are transformed to CSA masks, as seen in Table 1. Spatial connectivity patterns, based on e.g., 2D euclidean distances, are also supported. To this end, neuron positions can be defined either by neuron instance elements in the NeuroML file or sampled by a template distribution (e.g., Gaussian sampling). Synapse parameters such as weight and delay can be defined in analogously, by explicitly stating neuron instance parameters or by using distributions for whole layer connections. Currently, only Gaussian and Uniform distributions are defined, but additional distributions can be registered by sub-classing the Distribution class. In the case of region-to-region connectivity, atlas based connectivity is also supported. Additional details about the implementation of the different connectivity patterns can be found in the Supplementary Material.

After parsing, the layers and connections that make up a network are instantiated for the chosen simulator. First, the layers and neuron populations are translated to simulator specific instructions. If the simulator requires neuron positions, any distributions used are sampled at this point. Then, connections between layers are instantiated. Since this connection generation is computationally intensive, we use the Connection Generation Interface (CGI). The CGI calls available internal simulation engine functions to optimally generate connections instead of the high level calls through the Python interface (Djurfeldt et al., 2014). These native calls are typically more efficient (technical requirement 5). We use the libneurosim package, which supports the CGI and enables the generation of populations and connections in the simulator (i.e., NEST). Due to the modular nature of the implementation, individual components of ConGen can be easily replaced. For example, the C++ implementation of CSA (libcsa) offers increased performance over the Python implementation when generating connectivity, but has limited functionality. Thus, the Python implementation of CSA can be replaced by libcsa to accelerate the connectivity generation of large but simple networks.

For convenience to the users and in order to enable the simulation of the generated model in an specific framework, the ConGen back end contains a set of thin layer scripts which can call the target simulation engine (technical requirements 1, 2, and 3). Extending the ConGen back end to support new simulator engines is low effort and consists in the generation of a script which takes the connectivity objects, instantiates the model and, if desired, specifies simulator specific parameters. Pre- and post-processing of input and output data can also be added to this script by the user. At the moment the ConGen back end has a thin layer execution script for NEST and TVB.

The population and cell model parameters have to be defined by the user using simulator specific functions. This can be done either when the user imports the NeuroML file in his or her script or by modifying the thin layer simulator specific file in ConGen in order to add these parameters before model execution. In the current paper we focus specifically on connection generation as this is a complicated task on its own. Setting of model parameters could also be integrated into the ConGen front end, but is left as future work.

ConGen also allows the visual representation and generation of multiscale co-simulation models, and supports the output of multiscale configuration files. The orchestration and deployment of these multiscale simulations is complex (Klijn et al., 2019) and falls outside of the scope of the template based ConGen Translator simulation launching functionality.

## 3. Results

In this section we will describe first two use cases which are used to demonstrate the functionality of ConGen while addressing specific needs from the neuroscience community. Please refer to the Supplementary Material section to see where to find and how to execute the example files for these use cases. This section ends with an overview of the supported simulators.

### 3.1. Use Case 1: The Cortical Microcircuit Model

The Potjans and Diesmann microcircuit model (Potjans and Diesmann, 2014) is an abstraction of 1 mm^3^ volume of cortical tissue comprising four layers, each with one excitatory and one inhibitory population. The model has been used to address a variety of scientific questions and is able to show spiking dynamics similar to those observed in real cortical tissue. Due to its importance, we chose this model to test the whole functionality of ConGen, from visual language definition to simulation.

We constructed the model on two levels of abstraction: on the higher level, the representation of the column; on the lower, the representation of the single populations and their connection probabilities. It is important to note that ConGen provides the ability to define how the connectivity should be instantiated by the simulation on a probabilistic or deterministic way. By using CSA below the NeuroML description generated by ConGen, it is possible to create stable, portable and constant instantiations of connectivity patterns which will be the same independently of the target simulator. A step by step description of the model using ConGen is described in the following.

**Figure 6:**
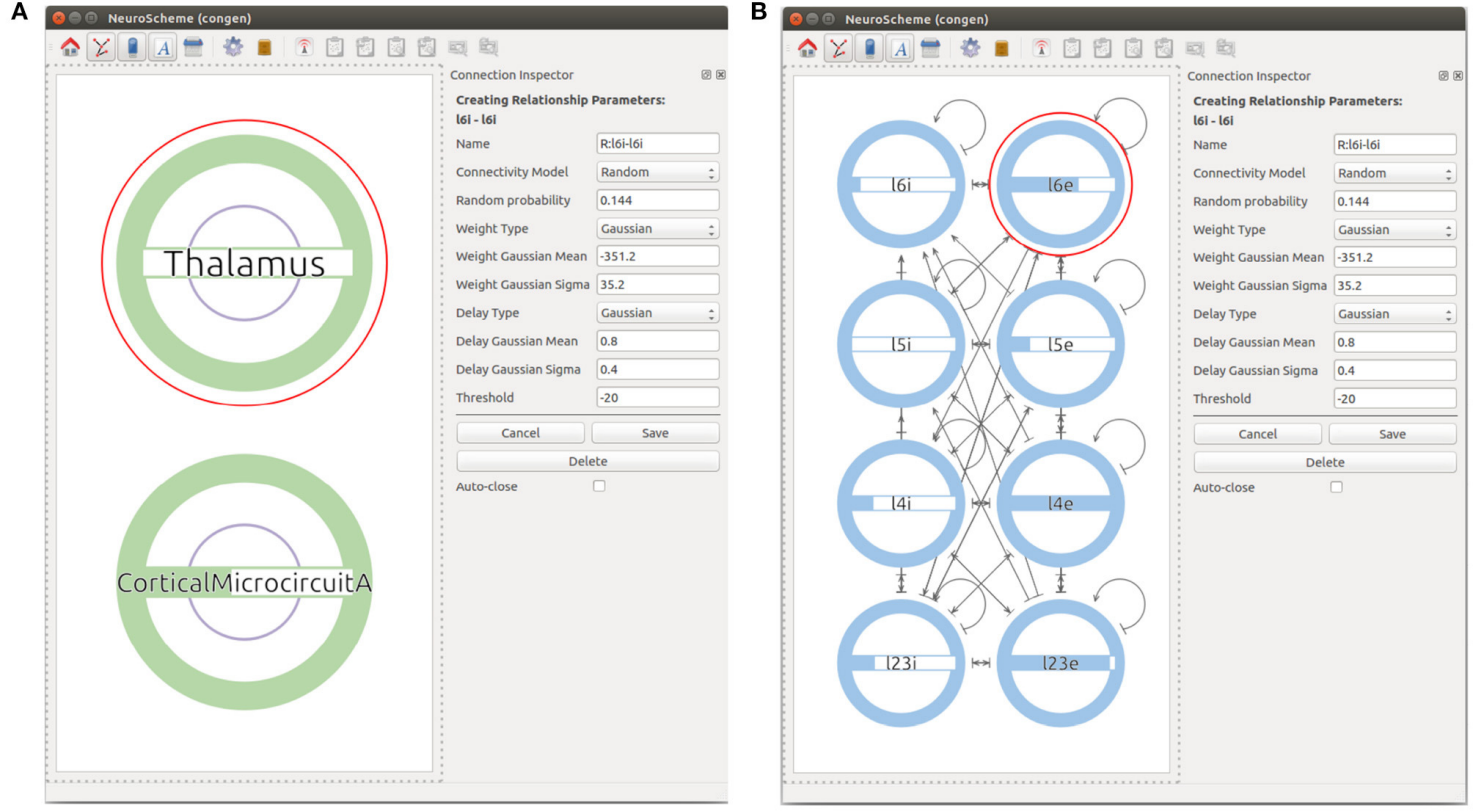
Views at different levels of cortical microcircuits. Panel **(A)** shows microcircuit super-population and the Thalamic super-population. Panel **(B)** presents the microcircuit super-population where all 8 populations can be seen with their respective connections.

First, the user can start by creating a super-population to represent the cortical microcircuit entity and the Thalamic region. The user can then go one level down in the visualization of the cortical microcircuit super-population to create the eight different populations of the cortical microcircuit using the *Add NeuronPop* option in the menu. After the eight populations have been created, the user can create connections between the populations by clicking and dragging the cursor from the source population to the target population. As the connectivity in the cortical microcircuit model is defined by a set of connection probabilities, the user defines a random connection with Gaussian distributions for the weights and the delays. In order to create an auto-connection, the user right-clicks on the desired population and selects *Add Auto Connection* from the menu. The model at this stage of creation is depicted in Figure 6. It is important to highlight that the connectivity in the original manuscript by Potjans and Diesmann (2014) is calculated under a specific set of considerations that are not reflected in the random distribution used in this use case. More specifically, the original model makes use of a fixed number of connections derived from the connection probability: *K*_*n, m*_ = ln(1−*P*_*n, m*_)/ln((*N*_*n*_*N*_*m*_−1)/*N*_*n*_*N*_*m*_), where *K*_*n, m*_ is the total number of connections between population *n* and population *m*, *P*_*n, m*_ is the connection probability between the two populations, and *N*_*x*_ denotes the number of neurons in population *x*. In our implementation, the connectivity of the model is generated using a pairwise Bernoulli distribution with probability *P*_*n, m*_. Therefore, variations in the actual number of connections between the model created with ConGen and the original model are to be expected.

Next, the user can create a set of input devices, in this case Poisson generators, in order to represent input arriving from other regions in the brain. This is done using the *Add Input* option and defining the frequency of the random stimuli produced by the Poisson generator. The input devices can be then connected using the click and drag operation from the source input to the target population.

**Figure 7:**
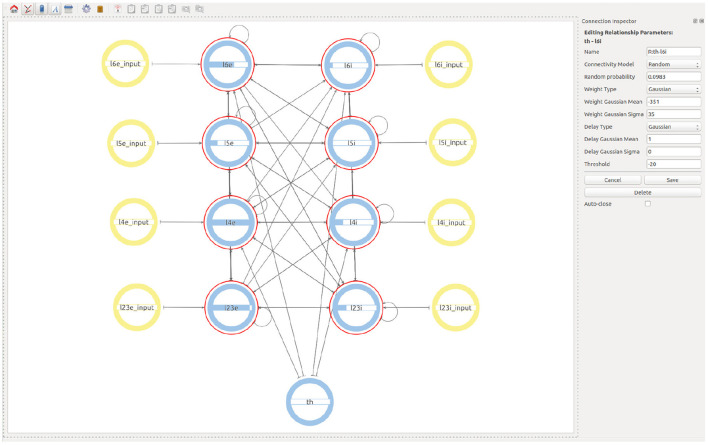
Final view of the complete model considering all populations, connections, and input devices.

Finally, in order to create the Thalamic connections, the user goes one level up in the visualization and then selects to expand the children of both the Thalamic and the microcircuit super-populations. This allows a Thalamic super-population to be created that can be then connected with the desired probability to the subpopulations representing the layer 4 and layer 6 of the microcolumn. See Figure 7 for the final version of the model.

The user can then export the resulting model to JSON or save as NeuroML, producing the file which can be then used by the generation back end to call NEST and execute the simulation. The time required by the ConGen backend to read, generate the model and create the connections using CSA is negligible compared to the actual connectivity generation step using CGI and the following execution of the model in NEST. This goes in agreement with the technical requirement 5 in section 2.2. An expert user is able to define the model in this use case in about 10 min. The resulting NeuroML file is easy to explore and understand by the users.

### 3.2. Use Case 2: Co-simulation of The Virtual Brain and NEST

The need to simulate the brain at different scales is an emerging requirement of modern computational neuroscience. Researchers may want to simulate the whole brain at a coarse resolution while simultaneously simulating specific areas that are relevant to answer a particular scientific question at a higher resolution. This interaction between simulators is complex (Klijn et al., 2019) and has been addressed in the past by several tools such as MUSIC (Djurfeldt et al., 2010). Having a common language to describe simulations which connects different scales and simulation back ends is essential for providing a usability layer to facilitate this ambitious next step in neuroscience. As ConGen's visual language is agnostic with respect to the target simulation platform, it can be used to define complex multiscale models for co-simulation.

In this second use case of ConGen we generate simulation scripts which are compatible with the co-simulation framework of the EBRAINS infrastructure developed by the Human Brain Project.^1^ In particular, we target a whole human brain co-simulation model where different parts are simulated at two scales using two simulators, The Virtual Brain (TVB; Sanz Leon et al., 2013) and NEST (Jordan et al., 2019). The coupling between the simulators is in part performed using the Elephant framework (Denker et al., 2018). For information going from NEST into TVB, the spike activity is translated into firing rates using Elephant. For information flowing from TVB into NEST, firing rates are tunneled to the NEST I/O back ends and defined as firing rates in heterogeneous Poisson generators. It is important to highlight that the coordination and deployment of the simulators is provided by external multiscale simulation infrastructure (Klijn et al., 2019) and not by ConGen itself.

ConGen is used to define the model and simulator at each scale, the connection points between simulators, and the translation modules to be used in order to transform data produced from one simulator and input to the other. In order to make it possible for the ConGen back end to identify which parts of the model belong to each scale, a prefix label is to be used for each component in the multiscale model. In this use case, we use “l” for all model components which should be simulated at the point neuron scale with NEST (in agreement with the model definition in use case 1) and the label “Brain_region” for all model components to be simulated at the whole scale level by TVB.

For the coarse scale, we divide the brain into 68 regions according to the Desikan Killiani cortical atlas (Desikan et al., 2006). Of the 68 regions, 67 are represented using a neural mass model, which in this case is the Kuramoto model (Kuramoto, 1975, 2003), and are to be simulated in TVB. The remaining region is represented as a cortical microcircuit as described in use case 1 and to be simulated in NEST. In this specific use case NEST will simulate a region in the atlas related to the auditory cortex on the right hemisphere, the right Transverse temporal cortex region. The model can be used to study the propagation of audio information from the auditory cortex to the rest of the brain and its interactions using simulations with sound stimuli. Please note that here for simplicity we assume that the phase represented by the state variable in the Kuramoto model can be linked to an indirect measure of the mean neural activity in the region and translated into spikes using the *Rate to Spike translator* available in the co-simulation framework.

**Figure 8:**
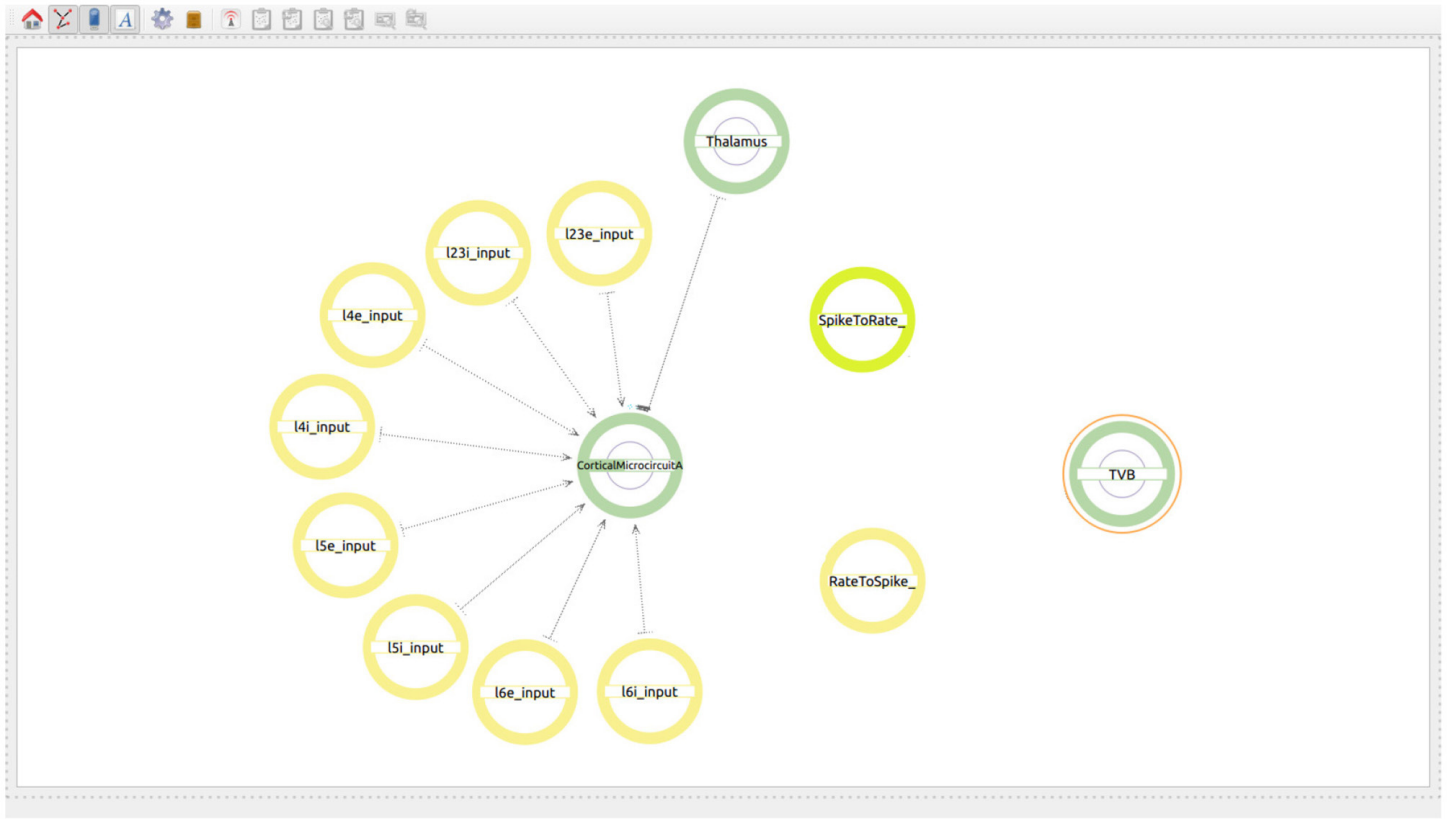
Modeling of the co-simulation use case starting with the super-population for the brain region represented by NEST, the super-population for the brain regions represented by TVB and the translator modules which have the task of translating spikes to rates and vice versa.

The user starts by generating two super-populations, one will represent the brain regions modeled in TVB and the other one the brain region modeled in NEST. Additionally, the user will create one spike to rate translator and a rate to spike translator (see Figure 8). These input devices are used to exchange information between scales and will be connected to specific populations within each super-population. Although obviously not existing in the real brain, translator components are nonetheless required to produce a functional multiscale co-simulation model.

Now the user can go one level down in the two super-populations. In the NEST region, the hierarchy, connectivity and inputs of the cortical microcircuit are defined as in use case 1. In the TVB super-population, 68 population elements are created. The neuron model to be used for 67 of the 68 regions will be a neuron mass model called “nmm kuramoto” corresponding to the Kuramoto model. The last region (correspond to ID 27) is modeled as a proxy for the NEST cortical microcolumn using the model called “proxy.” These elements are automatically numbered from 0 to 67 when created by ConGen and are linked to the correct region ID in the Desikan Kiliani brain atlas for simulation by the back end tool.

**Figure 9:**
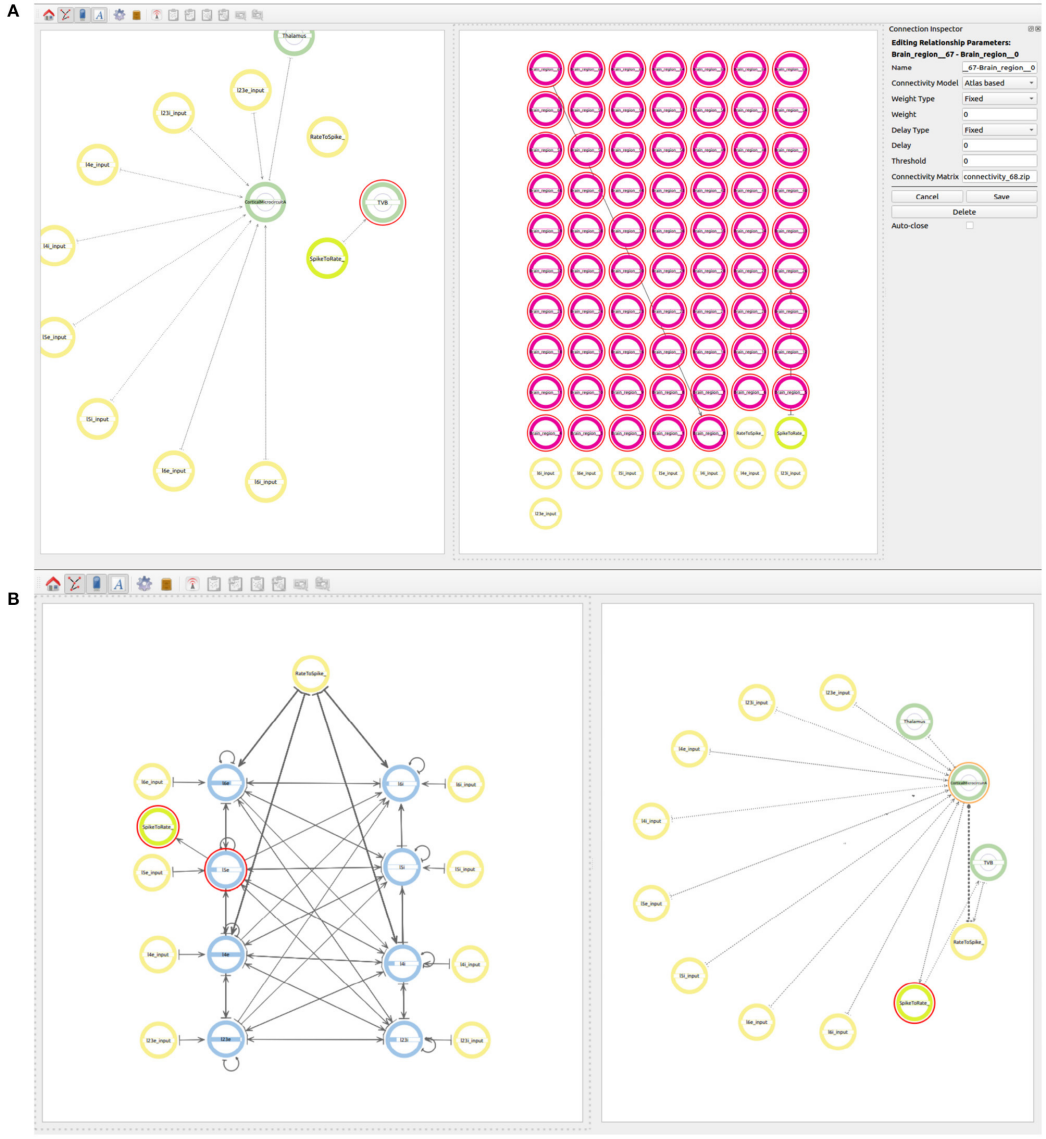
Views of the multiscale model. **(A)** Establishing connectivity within the brain regions in the TVB super-population using the Atlas based connectivity. A single connection from *Brain*_*region*_0 to *Brain*_*region*_67 is used to specify the atlas based connectivity. The connection from region 27 to the rate to spike translator device is also visible at this step. **(B)** The final whole connectivity setup visualized on the right and the inside view of the cortical microcircuit model on the left.

The next step is to define the connectivity between all the 68 regions. The type of connection to be used in the TVB models is *Atlas based* and the value in the *Connectivity Matrix* field should correspond to the connectivity matrix file to be used at simulation time (see Figure 9A). The value indicates a zip file which contains at least two files, one containing the weights matrix and another one containing the tract lengths matrix. The weights matrix is an *NxN* matrix which defines the strength of the connections between brain regions and where *N* is the number of regions in the specific parcellation to be used. The tract lengths matrix has the same dimensions as the weights matrix and specifies the distance between brain regions. These matrices are plain CSV files derived from empirical Diffusion Tensor Imaging (DTI) data [for more information please refer to Sanz Leon et al. (2013), section 1.1]. This ensures that the weight and the delay are loaded from the desired connectivity matrices before simulation. The user only needs to connect the first and the last region which will be connected with the desired atlas. It is important that the regions in the atlas match the range of regions selected in the model and that all regions involved are created with an index e.g., *Brain*_*region*_{*index*}.

In order to connect the two simulations on different scales, the output of all 67 regions in the TVB super-population, which are to be simulated in TVB, need to be connected to the *Rate to Spike translator* device created before. The output of the *Spike to rate translator* device must also be connected as input to the 67 regions in the TVB super-population. As mentioned before, region 27 serves as a *proxy* of the NEST super-population and, together with the translator modules, it is also used to simplify the exchange of information between both scales. As all regions in the TVB super-population are connected between each other using the atlas based connectivity, including region 27, it is only necessary to connect the output of region 27 to the *Rate to Spike translator* and the output of the *Spike to rate translator* as input to region 27. This way, the information exchange will be tunneled via the *proxy* region 27 in TVB.

Now the user can also connect the output of the *Rate to Spike translator* device as input to the excitatory and inhibitory populations in layers 4 and 6 of the cortical microcircuit model of the NEST super-population. The output of the excitatory population in layer 5 is then connected to the *Spike to rate translator* device (see Figure 9B).

Finally, the user can export the NeuroML file and execute the back end tool in order to generate the simulation files for TVB, NEST and the spike/rate translator modules. Using ConGen, and using the cortical circuit model as a starting point, the user takes about five additional minutes to specify the connectivity of this multiscale model. In return, the ConGen back end inputs the specific identifiers, connectivity patterns, and proxy interfaces in TVB, NEST, and the translator module files to enable co-simulation. The resulting files can then be executed using tools from the EBRAINS co-simulation framework (see the Supplementary Material section for more details). The performance of the connectivity generation is almost identical between having a single scale model or as part of a multiscale model. The only difference is in the connectivity to and from the translation modules, which depends on the co-simulation infrastructure.

### 3.3. Supported Simulation Engines

Even though the two use cases presented in this manuscript focus on NEST and TVB, ConGen can be easily extended to work with any simulator that supports the CGI. The only requirement is that a thin interfacing file needs to be generated to deal with importing, accessing the model data with the simulator-specific CGI commands, and launching the simulation. Simulation engines which support NeuroML can directly read the file generated by the ConGen interface and use it as a base for simulation. To summarize we provide an overview of the different simulators supported directly or via the diverse interfaces in Table 2.

**Table 2:** Simulator support by ConGen in different modalities: Connectivity setup and generation by ConGen back end, connectivity setup and basic simulator launching via ConGen back end, support for NeuroML file generated by ConGen front end using only standard features (see Supplementary Material for details on ConGen's extended connectivity features), and CGI connectivity generation through the ConGen back end.

**Type of support**	**NEST**	**TVB**	**EBRAINS multiscale co-simulation**	**All NeuroML compatible simulators**
Connectivity setup	YES	YES	YES	NO
Basic simulation launching	YES	YES	NO	NO
Standard NeuroML	YES	YES	YES	YES
CGI compatibility	YES	NO	NO	NO
Available use cases	YES	YES	YES	NO

## 4. Discussion

ConGen provides an easy way to generate networks at different scales, providing users the ability to visualize the relationships between scales in independent but correlated views and in side by side panels. As a concise but expressive visual language, ConGen provides a new way to define and navigate complex neural network models. The transcription of the defined circuits into NeuroML provides independence with respect to the ultimate choice of simulator.

The interaction offered by the ConGen's visual front end enables a rapid construction of neural network models through simple contextual menus, from defining a hierarchical structure with complex connectivity to parameterizing the neuronal and synaptic properties. The symbolic representations of the language synthesizes the most relevant features while eliminating less important details. The combination of schematic representations together with their arrangement in levels of abstraction yields simplified views of complex models.

The two use cases presented in this work illustrate the visual creation of connectivity in neural network models for their subsequent integration into simulation engines. Figure 7 provides a good example of how a user can examine the different levels of abstraction and easily identify the relationships between the components in the network. Use case 2 illustrates new possibilities to interact with abstractions that allow definition of multiscale models. At the higher level (Figure 8) we see the coarse components that form the model together with the abstract modeling components required to translate information between scales. Figure 9A shows an alternative view of the model, with the abstract high-level multiscale components on the left and the whole brain scale region definition on the right. In contrast, Figure 9B ConGen provides a more detailed view of the cortical microcircuit region and makes the relationship to translation and other components in the model easy to see and manipulate by the user. One important feature of the ConGen front end is the ability to have multiple panels concurrently showing different hierarchical levels of the model. These panels are connected between each other, so any actions done on one panel are automatically reflected on the others. This is useful for the exploration and design of multiscale models because it allows the user to visualize propagation of model changes from lower scales into higher scales as seen in Figure 9B.

Use case 2 provides an initial proof of concept for the definition of multiscale models compatible with the EBRAINS co-simulation infrastructure. The capabilities of the ConGen back end on this area are still limited and need to be extended to support further use cases and more complex interactions between simulators.

When comparing ConGen to existing simulator front ends, such as PyNN, further advantages become apparent. In PyNN, network creation code is inherently sequential, a characteristic that is contrary to the structure of a neuronal network. The visual language introduced in ConGen allows for a holistic view of a network model, which makes it easier to interpret the network or to spot errors. Other front ends like NEST Desktop (Spreizer et al., 2021), the TVB framework, and the NEURON graphic user interface (Carnevale and Hines, 2006) also allow the user to define networks and their connectivity in a visual way but are by definition, in contrast to ConGen, simulator-specific. We hope that ConGen can serve to decouple the way we create network models from the technical aspects of simulation, such as specific execution and deployment definitions, something that projects such as PyNN still require.

## 5. Conclusions and Future Work

ConGen addresses the complexity inherent to model generation in computational neuroscience from two perspectives. Firstly, it supports the visualization of complex models at different scales, allowing a reduction in the number of elements at higher scales and thus simplifying the visual complexity present in images with a high number of elements. Secondly, it reduces cognitive complexity by structuring the model in hierarchical levels of abstraction that summarize relevant features while eliminating less important details.

Multiscale modeling is a particularly demanding branch of computational neuroscience which exhibits a high degree of abstraction complexity. With the second use case provided in this manuscript we provide a proof of concept for a new visual approach to manage the complexity of constructing such models. The execution of multiscale models, which is not contemplated by the ConGen back end, has high computational demands which can only be efficiently fulfilled by using a dedicated framework such as the EBRAINS co-simulation infrastructure. Further extensions of ConGen may be required to fully facilitate current and future research in this field. With this in mind, ConGen was designed and implemented in a modular fashion; its integration with network definition standards allows developers and users to extend its functionality to include other simulation and emulation platforms (e.g., neuromorphic hardware like SpiNNaker; Furber et al., 2014) in the future.

The current version of ConGen does not fully constrain the modeler with regards to the types of connections it allows between network components scales. NeuroScheme has basic functionality to support this kind of aided connectivity generation but it would need additional metadata for each component to fully enable this functionality. For example, in the case of multiscale use cases, ConGen does not have any metadata which allows it to suggest or prevent possible connections to or from translators or devices. Adding such metadata would be a good extension for the future, and would further increase the usability for beginner users.

The next step for the ConGen back end is to update to NeuroML version 2 with LEMS. The network description of NeuroML version 2 has a different definition of the populations, which makes it necessary to describe cells within populations instead of providing generic cell types for all elements in a population. This is useful for small networks and morphologically detailed networks, but not suitable for the large scale networks targeted by ConGen. As discussed in the section 2, to allow generation of networks using the CSA we had to expand on the NeuroML interface. Although there is currently no direct way to port our work to NeuroML2, our next steps include working with the NeuroML2 development team in order to extend the language with at least a subset of the connectivity patterns available in CSA such as all-to-all or one-to-one. This will probably be implemented with a new population description using LEMS. Another alternative is to move toward NeuroMLite (https://github.com/NeuroML/NeuroMLlite) which is still in development but seems to move toward standardized description of biological and artificial networks with features which are compatible with the ConGen goals and architecture.

Future work also involves extending the back end to incorporate more cases for different simulators and allow more complex models, especially for co-simulation. Plasticity is also an important feature of connectivity that may require new visual language concepts. The direct next extension of ConGen is to allow plastic synapses to be defined and to implement an interactive loop (see Figure 5), the dashed arrow indicates the transport of simulation results back to ConGen) where connectivity can be refreshed based on data produced during simulation. This new step will provide a new graphic interface to study dynamic changes in the connectivity of large scale networks.

PyNN has evolved as a strong domain specific language for network representation in the last years. Future work will also involve extending NeuroScheme and the back end in order to support PyNN as a description language. This can be achieved through the porting to NeuroML version 2. The automatic benefit here is that PyNN already incorporates CSA in its description and an extension will increase the range of potential target simulators which can benefit from the visual language proposed by ConGen. Adding models generated by the ConGen front end to Open Source Brain (Gleeson et al., 2019) would also be a step forward to increase integration with current efforts in the direction of standardization. Additionally, the ConGen back end could be later integrated into Open Source Brain, thanks to their usage of common standard like NeuroML and PyNN.

In summary, with this work we propose a novel simulator-agnostic method for the definition and generation of connectivity in multiscale neural network models. ConGen also represents a new way to generate models which can be ported to different simulators using NeuroML or the ConGen back end in order to perform benchmarking and compare functional and execution metrics between simulation engines at different scales. Using the ConGen framework does not require any programming experience; any scientist, regardless of background, can employ a common visual language to express, share, study, and implement connectivity for *in-silico* experimentation, in order to solve complex questions regarding the relationships between structure and function in the brain.

## Data Availability Statement

The original contributions presented in the study are included in the article/Supplementary Material, further inquiries can be directed to the corresponding author/s.

## Author Contributions

OR, SM, PT, LP, AP, AM, SD-P, and WK worked on the design of the system. IC, OR, SM, PT, and LP worked on the design of the visual front end. PH, AP, AM, SD-P, and WK worked on the rest of the workflow and designed the use cases. PH, IC, WK, and SD-P worked on the implementation. All authors conceived of the project, reviewed, contributed, and approved the final version of the manuscript.

## Funding

The research leading to these results has received funding from the Spanish Ministry of Economy and Competitiveness under grants C080020-09 (Cajal Blue Brain Project, Spanish partner of the Blue Brain Project initiative from EPFL), TIN2017-83132, PID2020-113013RB-C21, and PID2020-113013RB-C22, as well as from the European Union's Horizon 2020 Framework Programme for Research and Innovation under the Specific Grant Agreements No. 785907 (Human Brain Project SGA2) and 945539 (Human Brain Project SGA3). This research has also been partially funded by the Helmholtz Association through the Helmholtz Portfolio Theme Supercomputing and Modeling for the Human Brain.

## Conflict of Interest

The authors declare that the research was conducted in the absence of any commercial or financial relationships that could be construed as a potential conflict of interest.

## Publisher's Note

All claims expressed in this article are solely those of the authors and do not necessarily represent those of their affiliated organizations, or those of the publisher, the editors and the reviewers. Any product that may be evaluated in this article, or claim that may be made by its manufacturer, is not guaranteed or endorsed by the publisher.

## Footnotes

^1^ https://ebrains.eu/
