# Supplementary material for: ConGen—A Simulator-Agnostic Visual Language for Definition and Generation of Connectivity in Large and Multiscale Neural Networks
Source: Front Neuroinform. 2022 Jan 7;15:766697. doi: 10.3389/fninf.2021.766697 (PMC8777257; doi:10.3389/fninf.2021.766697)
Supplement: Supplementary file 1 [file Data_Sheet_1.PDF]

1 Supplementary material

1.1 Architecture of the ConGen backend

Python Module Structure All of the python files are contained in a package named `vicogen`. The NeuroML files are handled in the `neuroml` subpackage. The modules in the `neuroml` package are intended to hold the data structures of the translation and parsing NeuroML files. The module `parsing.py` contains all the functions for parsing NeuroML files into the data structures. The `vicogen` package itself contains a few different modules:

- `_vicogen.py` contains functions for accessing the file parsing from the `neuroml` package, starting simulations with NEST and printing connectivity matrices and lists to the console or to file. All of the functions in this module are made available on a package level.
- `_nest.py` is the module responsible for connecting the translator to the simulator NEST. It provides functions for translating populations and calling the CGI-Functions for NEST, and also starting simulations.
- `_tvb.py` is the module responsible for connecting the translator to TVB.
- `__main__.py` is a required file for using the package by itself on a command line. It allows the package to be called like a module using `python -m ConGen PARAMETERS`.
- `commands.py` provides the command arguments that can be used when calling the module from a command line.
- `__init__.py` is called when `vicogen` is imported as a python package. It gathers all the functions from the other modules and provides them on a package level.

The `vicogen` package can be used either as a normal python package using `import vicogen` or executed as a python file with command lines for quick access (See supplementary material in Section 3).

The modules in the `vicogen` package are written to ensure minimum dependencies between each other. For example, new simulators can be added easily as all references to NEST and TVB are contained in the `nest.py` and `tvb.py` modules respectively].

Class Structure To represent NeuroML in Python in a way that is extensible and maintainable, the class structure makes use of inheritance and "duck typing". For example, all connectivity pattern classes are subclasses of the `ConnectivityPattern` class, which has an empty function `mask()` that all subclasses have to implement. This makes it easier for developers to see which functions they need to implement to add a new pattern. Fig. 1 shows a class diagram of all the classes represented in the `neuroml` package. All connectivity patterns are subclasses of `ConnectivityPattern` and `Projection`. Adding a new connectivity pattern can be done by creating a new subclass of `ConnectivityPattern` and assigning a CSA mask to `self._mask` in the `__init__` method. Similarly, new subclasses can be created to add new distributions, neuron position functions, and inputs.

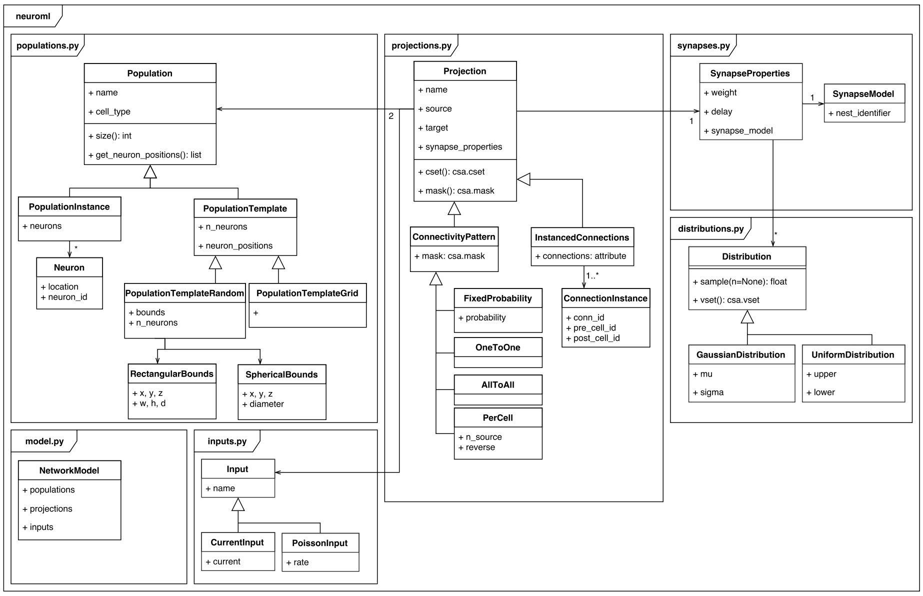

Figure 1: Class diagram of the `neuroml` package.

1.1.1 Changes to NeuroML

Some additions and changes had to be made to the NeuroML XSD schema for our use cases. All additions and changes are listed and described here.

One-To-One Connectivity Of the common elementary connectivity patterns, NeuroML is missing the One-To-One connectivity. Since a one-to-one connection does not need any parameters, adding it to the XSD Scheme is trivial:

```
<xs:element name="one_to_one">
    <xs:complexType/>
</xs:element>
```

While the One-to-One connectivity may not be common for biological networks, it is important for connecting inputs and recording devices.

Atlas based Connectivity The atlas based connectivity allows users to specify connectivity between a set of nodes using data in the form of a connectivity matrix. The parameter connectivity_matrix indicates the name of a zip file which includes the weights and the tract_lengths in form of CVS files. Both the weights and the tract_lengths data structures are NxN matrices where N is the number of nodes to be connected.

The atlas based connectivity is described in the XSD Scheme as follows:

```
<xs:element name="atlas_based">
    <xs:complexType>
        <xs:attribute name="connectivity_matrix" type="xs:string"/>
    </xs:complexType>
</xs:element>
```

Input Sites In NeuroML, an input is connected to a population by defining an input site. The sites function the same way as projections, as they can connect an input to a population using either a connectivity pattern or connection instances. Since using an input site is the same as connecting an input through a projection, this creates an unnecessary redundancy in the file structure. New connectivity patterns would have to be created for projections as well as for inputs. To avoid the redundancy and additional workload, input sites are not supported by the parser. Instead, inputs are connected the same way two populations would be connected: With a projection where the source is interpreted as a population with size 1. This makes the file format easier to maintain and expand, and also simplifies the parser.

Spatial Connectivity In order to be able to support spatial connectivity, being Gaussian the most used at network scale neuroscience, two new XML structures for Gaussian spatial connectivity in 2D and 3D have been added to the NeuroML file format.

```
<xs:element name="gaussian_connectivity_2d">
    <xs:complexType>
        <xs:attribute name="sigma" type="xs:decimal"/>
        <xs:attribute name="cutoff" type="meta:NonNegativeDouble"/>
    </xs:complexType>
</xs:element>
<xs:element name="gaussian_connectivity_3d">
    <xs:complexType>
        <xs:attribute name="sigma" type="xs:decimal"/>
        <xs:attribute name="cutoff" type="meta:NonNegativeDouble"/>
    </xs:complexType>
</xs:element>
```

Both XML elements have the parameters `sigma` and `cutoff`. The `sigma` parameter controls the size of the Gaussian that is used for calculating the probability of a connection. The `cutoff` parameter can be used to set the maximum distance a point can have to the center of the Gaussian. With this addition to the XML structure, a Gaussian spatial connectivity can now be given as follows:

```
<connectivity_pattern>
    <gaussian_connectivity_2d sigma="1.5" cutoff="3"/>
</connectivity_pattern>
```

The positions of the neurons can already be defined in NeuroML by either giving a position element to all neuron instances, or by adding a <pop_location> element to a template based population. The neuron locations in NeuroML are always three dimensional. When using 2D spatial connectivity, the value for the `z` dimension is ignored.

Adding these two structures provides simple spatial connectivity for 2D and 3D populations, but more advanced forms of spatial connectivity are still missing. Additional features may be scaling and translating of neuron positions, or even generic projection functions. Generic projection functions are supported in CSA, but have no equivalent in NeuroML.

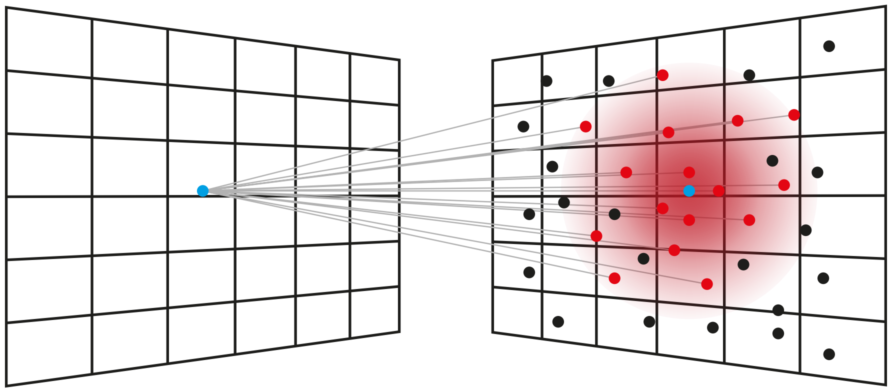

Figure 2: Gaussian spatial connectivity on two 2D-Layers. The neuron to connect from the source layer gets projected onto the target layer using an arbitrary mapping function (blue dots). The distance metric from CSA gets applied to a Gaussian sampler (red circle), resulting in Gaussian local connectivity (red dots).

Distributed Synaptic Parameters When describing connectivity parameters, e.g. synaptic weights or delays, NeuroML only provides static values. Only instanced connections can have individual parameter values. For connectivity patterns though, NeuroML provides no way of giving each connection in the connectivity pattern a different value. All connections in the connection pattern share the same static value for their parameters. A solution to this is using distributions for parameters. To accomplish this, a new `DistributedProperty` complex type has been added to the XSD Scheme. A distributed property can hold one of the defined distributions: `GaussianDistribution` and `UniformDistribution`.

```
<xs:complexType name="DistributedProperty">
    <xs:choice>
        <xs:element name="GaussianDistribution" type="GaussianDistribution"/>
        <xs:element name="UniformDistribution" type="UniformDistribution"/>
    </xs:choice>
</xs:complexType>

<xs:complexType name="GaussianDistribution">
    <xs:attribute name="center" type="xs:decimal" use="required"/>
    <xs:attribute name="deviation" type="xs:decimal" use="required"/>
</xs:complexType>

<xs:complexType name="UniformDistribution">
    <xs:attribute name="lower" type="xs:decimal" use="required"/>
    <xs:attribute name="upper" type="xs:decimal" use="required"/>
</xs:complexType>
```

A distributed property can be defined inside a `synapse_props` block of a projection. So far, only *weight* and *delay* are supported, as the CGI implementation in the `libneurosim` package only supports weight and delay parameter for now.

```
<xs:all minOccurs="0">
    <xs:element name="weight" type="DistributedProperty"/>
    <xs:element name="delay" type="DistributedProperty"/>
</xs:all>
```

The Gaussian distribution defines a center and a deviation, which correspond to μ and σ of a Gaussian. The uniform distribution has an upper and lower bound and gives an even probability for every value between these bounds. Adding a new distribution can be done in XSD by adding it to the `DistributedProperty` complex type. New types of distributed parameters can be added in the `SynapseInternalProperties` complex type as a `DistributedProperty`. An example for distributed parameters as child elements in a NeuroML file can be seen here:

```
<synapse_props synapse_type="StaticSynapse" threshold="-20">
    <weight>
        <GaussianDistribution center="87.8" deviation="8.8"/>
    </weight>
    <internal_delay>
        <UniformDistribution lower="0.5" upper="1.5"/>
    </internal_delay>
</synapse_props>
```

The distributed properties are entirely optional and synapse properties can still be defined as an attribute of `<synapse_props>` instead of a child element. Alternatively, a static value may also be given as a child element of `synapse_props` for weight and delay, making it a possible replacement for the old way of writing parameters. It would have been possible to encode the distributed parameters in the string attributes of the `synapse_props` element, which would have required no changes to the XSD, but this would also make it impossible to check the validity of the elements using XSD. Additionally, encoding distributions as a string would introduce a structural inconsistency to the NeuroML format which would make it difficult for parsers to correctly parse the NeuroML files.

These NeuroML extensions were necessary as NeuroML's connectivity definitions for large networks is limited. Dynamically creating connectivity patterns similar to CSA is not possible in NeuroML, and distributed properties are not supported. The only way to recreate spatial connectivity or distributed synapse parameters is by computing the patterns or distributions before writing the NeuroML file, and using NeuroML's connection instances instead. This would, especially for large networks, increase the file size of the NeuroML files dramatically. NineML does inherit these problems, although it does support distributed parameters.

2 Installation Instructions

2.1 CSA and NEST with CGI

1. Install autoconf using `sudo apt-get install autoconf`

2. Clone and install libneurosim:

```
git clone https://github.com/INCF/libneurosim.git
cd libneurosim
./autogen.sh
./configure --prefix={libneurosim_installpath}
make
make install
```

3. Install csa:

```
git clone https://github.com/INCF/csa.git
cd csa
./autogen.sh
./configure --with-libneurosim={libneurosim_installpath}
  --prefix={csa_installpath}
make
sudo make install
```

4. Install NEST:

```
git clone https://github.com/nest/nest-simulator
cd nest-simulator
cmake .. -Dwith-libneurosim={libneurosim_installpath}
  -Dwith-ltdl
make
sudo make install
```

5. Install TVB:

```
Please follow the instructions in:
$https://www.thevirtualbrain.org/tvb/zwei/brainsimulator-software?\_ga=2.1
```

6. Install NeuroScheme: The version of the ConGen domain in NeuroScheme used for this manuscript can be found in this repository under the congen branch: https://github.com/multiscale-cosim/NeuroScheme.git

   Installation instructions can be found in the repository as well.

2.1.1 Starting ConGen

To start the interactive connectivity generation, use `./NeuroScheme -d congen`. To run the backend

2.2 Installing the ConGen backend

The ConGen back end code and the files used for the different use cases can be found in this repository: https://github.com/multiscale-cosim/ConGen.git

The Python package does not need to be installed to be used, but can be added to your Python libraries. In the ConGen root, execute:

```
mkdir build && cd build
cmake ..
make
sudo make install
```

2.2.1 Environment configuration

Add nest-simulator site-packages, vicogen, vicogen/neuroml and csa installation folders to $PYTHONPATH.

3 ConGen backend usage instructions

ConGen backend can be used independently of the GUI. The ConGen package can be imported in Python using `import vicogen`. Alternatively, ConGen can be used directly from the console by executing it as a Python module in the command line.

```
python -m vicogen [-h] [-o [OUTFILE]] [-t SIMTIME] [-c]
                  [-d] [-v] [-m multiscale]
                  [-b simulate_with_tvb]
                  [-n simulate_with_nest]
                  [--nest-options NEST_OPTIONS]
                  [-l multiscale_labels]
                  modelfile
```

`modelfile` is the NeuroML file to be parsed and has to be given. The module supports the following command line arguments:

To run and simulate for 100ms the use case 1 files provided in the ConGen repository execute:

```
python3 -m vicogen -v -n True -t 100
  {Path_to_ConGen}/ConGen/Frontiers2021manuscript/
  nml/pd_final.xml
```

To generate the Cosimulation files for a simple example in the repository execute:

```
python3 -m vicogen -m True -n True -b True -v
  {Path_to_ConGen}/ConGen/Frontiers2021manuscript/
  nml/UC3_simple_multiscale.xml
```

To generate the Cosimulation files for the second use case files in the repository execute:

```
python3 -m vicogen -m True -n True -b True -v
  {Path_to_ConGen}/ConGen/Frontiers2021manuscript/
  nml/UC2_final.xml
```

**Argument**	**Description**
`-h, --help`	Shows a help message on the usage.
`-o [OUTFILE], --outfile [OUTFILE]`	Set the file to write output to. If not given, the output is written to `stdout`.
`-t SIMTIME, --SIMTIME`	Simulate the model for a given amount of milliseconds.
`-c, --write-connections`	Instead of simulating the network, parse the connections and write them to output.
`-n, --nest`	Simulate with NEST.
`--nest-options NEST_OPTIONS`	Additional options for NEST.
`-b, --tvb`	Simulate with TVB.
`-m, --multiscale`	Generate EBRAINS co-simulation scripts for multiscale models.
`-l, --multiscale-labels`	Labels to split the NeuroML model into scales.
`-d, --debug`	Print debugging information.
`-v, --verbose`	Print verbose messages.

4 ConGen supported cell types

The currently supported cell types in the ConGen front end are: "iaf psc alpha", "cell", "cell2CaPools", "baseCell", "iafTauCell", "iafTauRefCell", "iafCell", "iafRefCell", "izhikevichCell", "izhikevich2007Cell", "adExIaFCell", "fitzHugh-NagumoCell", "fitzHughNagumo1969Cell", "pinskyRinzelCA3Cell", "nmm_kuramoto", "nmm_2doscillator", "proxy" and "undefined".
